# Miliary coccidioidomycosis mimicking tuberculosis: Case report and review of literature

**DOI:** 10.1016/j.mmcr.2024.100668

**Published:** 2024-09-13

**Authors:** Oscar E. Gallardo-Huizar, Joyce Lee, Kailyn Kim, Arthur C. Jeng

**Affiliations:** a Department of Internal Medicine, Olive View-UCLA Medical Center, Sylmar, CA, USA; b David Geffen School of Medicine, University of California, Los Angeles, CA, USA; c Department of Infectious Disease, Olive View Medical Center, Sylmar, CA, USA

**Keywords:** Coccidioidomycosis, Miliary coccidioidomycosis, Tuberculosis (TB), Latent tuberculosis infection (LTBI), Immunoglobulin G (IgG) and Immunoglobulin M (IgM)

## Abstract

Miliary coccidioidomycosis is a severe manifestation of diseases caused by *Coccidioides immitis* and *Coccidioides posadasii* that is endemic to the southwestern United States as well as Central and South America. While most cases of coccidioidomycosis present with pulmonary disease, certain risk factors increase the risk for disseminated disease. We present a case of miliary coccidioidomycosis in a 46-year-old patient with uncontrolled diabetes. Additionally, we review the features of thirty-seven cases of patients with miliary coccidioidomycosis.

## 1 Introduction

Coccidioidomycosis is a disease caused by the dimorphic fungi *Coccidioides immitis* and *Coccidioides posadasii* that is endemic to the southwestern United States, Washington State, as well as Central and South America [1,2]. Initial infection occurs predominantly by inhalation of aerosolized arthroconidia and rarely by direct cutaneous inoculation [3,4]. The main risk factor for its infection is living, working, or traveling in an area where *Coccidioides* is endemic [5]. Immunocompromised persons, especially those with T-lymphocyte impairment or human immunodeficiency virus (HIV) infection, pregnant women (particularly in their third trimesters and immediately postpartum), and individuals with diabetes mellitus are at the highest risk of infection [6,7]. The disease can be divided into three general categories: primary pulmonary infection (most common), extrapulmonary dissemination, and rarely, primary cutaneous coccidioidomycosis [8].

Disseminated coccidioidomycosis is rare but can manifest as a miliary pattern with chest imaging. Primary pulmonary and disseminated disease presents with symptoms similar to other respiratory illnesses; therefore, it is important to have a high index of suspicion for patients living or with recent travel to areas where *Coccidiodes* is endemic [9,10].

Here we present a case of miliary coccidioidomycosis, with an initial presentation mimicking miliary tuberculosis, which was diagnosed through positive IgM and IgG serologies. Furthermore, we describe the literature on miliary coccidioidomycosis in both immunocompromised and immunocompetent individuals.

## 2 Case presentation

A 46-year-old Hispanic female with a reported history of treated latent tuberculosis infection (LTBI), childhood polio, uncontrolled diabetes mellitus (A1c 12.9 %), androgen insensitivity syndrome (genotype XY, underwent bilateral orchiectomy in 2011 complicated by incidental seminoma), presented with 3 weeks of dry cough, shortness of breath, unintentional 25-pound weight loss, and headache (day 0). The patient stated that her symptoms started after driving through the “California Valley”. On presentation (day 21), she was afebrile with a heart rate of 98, tachypneic to a respiratory rate of 28 with an oxygen saturation of 86 % on room air. Physical exam on admission was notable for diaphoresis and unremarkable lung exam.

Initial laboratory results were significant for normocytic anemia without leukocytosis (Hgb 11.4 g/dL) with chemistry showing hyponatremia (Na 126 mmol/L), non-anion gap respiratory alkalosis (pH 7.48, CO2 37 mmol/L), and elevated lactate (3.2 mmol/L). Further evaluation of the patient's persistent hyponatremia revealed a high urine osmolality (427 mOsm/kg) and urine sodium of 108 mmol/L.

Initial chest radiograph was notable for a miliary pattern in the lungs, with airspace disease in the left upper lung zone (Figure A). A follow-up computed tomography (CT) (Figure B) scan of her thorax without contrast demonstrated diffuse bronchocentric-prominent nodularity, left upper lobe fibro-cavitary disease, and a few calcified granulomas. Given her recent travel history, *Coccidioides* serology studies (IgM and IgG), complement fixation, and identification were sent and were positive (IgM 0.774, IgG 1.811, complement fixation 1:16).

**Figure A:**
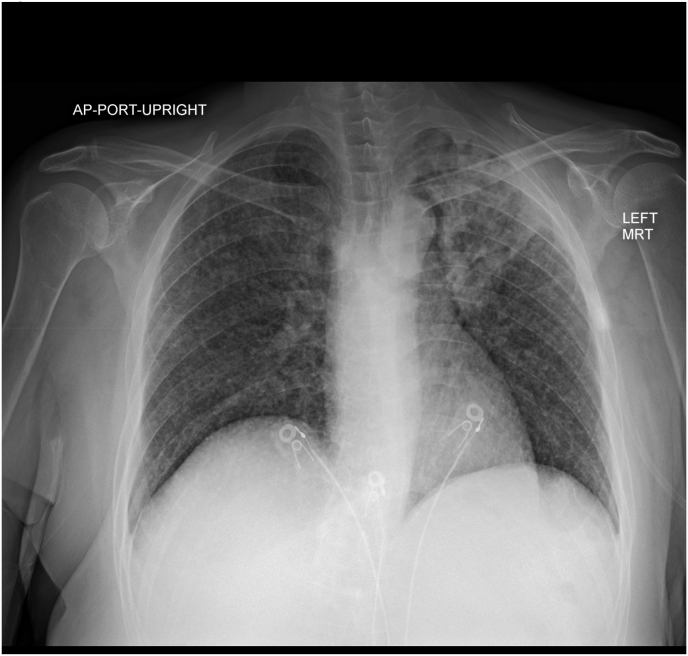
Miliary pattern in the lungs with airspace disease in the left upper lung zone.

**Figure B:**
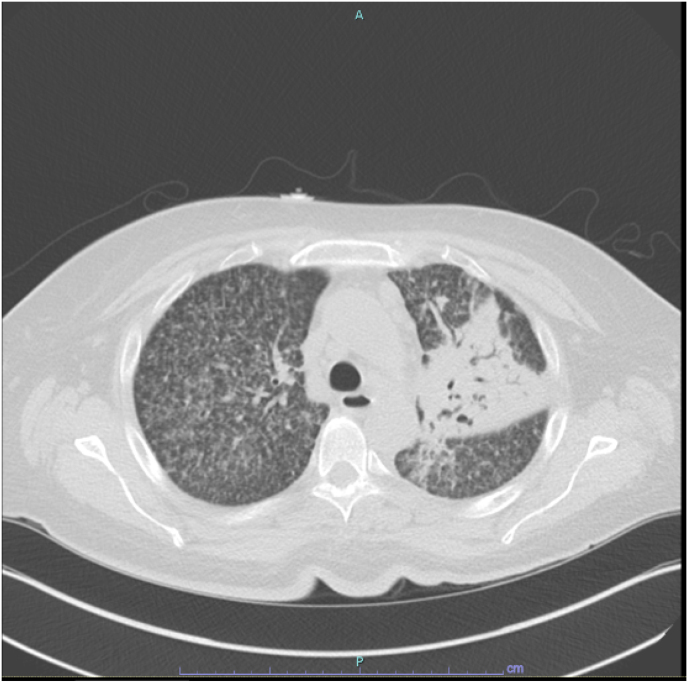
diffuse bronchocentric-prominent nodularity and left upper lobe fibro-cavitary disease.

Acid-fast bacilli sputum cultures were unremarkable (day 24), and a QuantiFERON tuberculosis test was negative. Initial blood and sputum (fungal included) cultures did not show any pathogens and urine cultures demonstrated 35,000 CFU/mL *Streptococcus agalactiae*.

The patient was started on fluconazole 600 mg daily and salt tablets for the suspected syndrome of inappropriate diuretic hormone secretion (SIADH) secondary to pulmonary disease (day 25). Studies were negative for tuberculosis (described above), and legionella and histoplasmosis screening (urine antigen) was also negative. Risk of Sarcoidosis was low, so no specialized testing was obtained based on radiographic findings. She remained hospitalized for persistent hypoxia secondary to pulmonary disease and treatment of hyponatremia up until her discharge 11 days later after significant clinical improvement (day 32). On outpatient follow-up a month later (day 62), the patient was doing well and adherent to her fluconazole treatment, and detailed plans were made to repeat bloodwork, re-check *Coccidioides* titers, and obtain a CT chest to check for a clinical resolution. Unfortunately, she was lost to follow up shortly after that.

## 3 Discussion

Coccidioidomycosis is a relatively common infection. During 2000–2018, a total of 65,438 coccidioidomycosis cases were reported in California, with a median age-adjusted annual incidence of 7.9 per 100,000 population [11]. Exposure to *Coccidioides* generally results in asymptomatic infection [12]. Disseminated coccidioidomycosis, when the fungus spreads from the lungs, can present with a broad spectrum of clinical manifestations, but the fungus likes to infect the bones (especially the spine), joints, skin, meninges, and lymph nodes involving the neck. Immunosuppressed individuals living in endemic areas are at increased risk of disseminated disease [13,14].

The patient described in our case had uncontrolled diabetes as the main risk factor for disseminated disease. In our review of the literature, the most common comorbidities associated with miliary disease were diabetes (18 %), immunosuppression (33 %), and pregnancy (12 %) (Table 1, Table 2). The category of “immunosuppression,” consisted primarily of HIV/AIDS, malignancy, transplantation, and use of immunosuppressive medications, factors which involved deficits in cell-mediated immunity. There was also a predominance of males (79 %). Most of the miliary patterns seen on imaging were not accompanied by other radiological findings such as cavitation or consolidation, reflecting that this disseminated finding is not from a bronchopulmonary etiology or spread, but rather from the bloodstream seeding the pulmonary capillary beds [15]. It is of interest that our case had both miliary nodularity as well as a left upper lobe cavitary lesion. Among the cases reviewed, most of the patients that were treated with an anti-fungal received amphotericin B (89 %), likely because of severe disease and/or because oral azoles were not available at the time, as 58 % of cases were reported before the year 2000. However, fluconazole was also used commonly (33 %) in those treated, either as monotherapy or in conjunction with amphotericin. Thirty percent (8/27) of patients received steroids as adjunctive therapy to the anti-fungal, of which 38 % (3/8) of these had clinical improvement and 62 % (5/8) died. It is difficult to ascertain the role and/or value of steroids, which were given to decrease the inflammation but could potentially cause further immunosuppression, from these case reports. It is also likely that the more severe cases received steroids, which may lead to higher mortality being reported in the literature. However, it is notable that those who did not get anti-fungal treatment and the 3 patients who received steroids only, all died, underscoring the life-threatening severity of miliary coccidioidomycosis.

**Table 1:** Reported cases of miliary coccidioidomycosis in adults.

Author and Year	Age/Gender/Race	Comorbidities Present at Diagnosis (Co-existing Dx)	Diagnosis	Initial Chest Radiograph	Treatment	Outcome
[21]	37, F, Caucasian	Pregnant, myelofibrosis, on steroid therapy	Autopsy, histopathology, cultures	Fine miliary densities	None	Death
[22]	55, M, White	None	Serum serology, serum complement fixation.	Bilateral, 1–2mm nodular infiltrates distributed evenly throughout lung fields	IV and intrathecal amphotericin B	Alive
			Definitive dx w/urine and sputum cultures			
[23]	35, M, Caucasian	Diabetes	Precipitin antibody C. immitis	Miliary pattern and pleural effusion	Amphotericin	Death
[24]	23, M, White	None	Sputum cultures, serum complement fixation	Bilateral patchy areas of consolidation; multiple nodular radiopacities	IV amphotericin B, oral ketoconazole	Alive (“not returned to his usual state of health”)
[24]	26, M, White	None	Sputum cultures, serum complement fixation	Bilateral patchy areas of consolidation; multiple nodular radiopacities	IV amphotericin B	Alive (“has returned to his usual state of health”)
[25]	83, M, White	Chronic lymphocytic lymphoma	Blood culture	Miliary	Dexamethasone 24 mg	Death (one month later)
[25]	20, M, Mexican American	Renal Transplant	Blood culture	Miliary with LLL infiltrate	Amphotericin B 590mg, and Prednisone 25mg	Alive (one month later)
[25]	81, M, White	Chronic lymphocytic leukemia	Blood culture	Miliary	Amphotericin B 132 mg, Prednisone 10mg	Death (one month later)
[25]	54, F, White	Breast carcinoma, Chronic lung disease	Blood culture	Miliary and LUL cavitary lesions	Amphotericin B (300mg), Prednisone 5 mg	Death (one month later)
[25]	68, M, White	Large cell carcinoma of the lung, squamous cell carcinoma of the hypopharynx	Blood culture	Miliary	Dexamethasone (16mg)	Death (one month later)
[25]	24, M, White	AIDS	Blood culture	Miliary	Amphotericin B (1600mg), ketoconazole	Alive (one month later)
[25]	62, M, White	Chronic lung disease, idiopathic cardiomyopathy	Blood culture	Miliary	Amphotericin B (1040 mg), prednisone 25mg	Death (one month later)
[25]	71, M, White	Glioblastoma	Blood culture	Miliary	Amphotericin B (155mg), dexamethasone 12 mg	Death (one month later)
[25]	32, M, Black	AIDS	Blood culture	Miliary	Ketoconazole	Death (one month later)
[9]	38, M, African American	Hypertension, CHF, cocaine abuse, EtOH, tobacco	Complement fixation	RML infiltrate (on initial chest radiograph), subsequent imaging with miliary pattern	Fluconazole	Death
[9]	23, M, Caucasian	IVDA, tobacco, EtOH	Complement fixation	Miliary pattern	Fluconazole and amphotericin	Death
[9]	65, M, Caucasian	None	Complement fixation	Miliary pattern	Fluconazole and amphotericin	Alive
[9]	41, F, Caucasian	IVDA, tobacco	Complement fixation	Miliary pattern	Fluconazole	Alive
[9]	26, M, African American	None	Complement fixation	Miliary pattern	Fluconazole, amphotericin, interferon	Death
[9]	34, M, Caucasian	None	Complement fixation	LL lobar infiltrate (on initial chest radiograph), subsequent imaging with miliary pattern	Amphotericin	Alive
[9]	25, F, Hispanic	Pregnant	Complement fixation	Miliary pattern	amphotericin	Alive
[9]	27, F, Hispanic	Pregnant	Complement fixation	Miliary pattern	Fluconazole and amphotericin	Alive
[26]	21, F, Hispanic	Gestational Diabetes and Pregnancy	Blood cultures, complement fixation	Diffuse miliary infiltrates	Amphotericin, fluconazole, steroids	Clinical Improvement
[27]	31, M, Korean	adrenoleukodystrophy	skin biopsy with spherules	Miliary infiltrates	Amphotericin, fluconazole	Clinical resolution
[28]	78, M, Caucasian	Rheumatoid Arthritis on Infliximab therapy	BAL, endospores visualized	Bilateral miliary interstitial infiltrate pattern	amphotericin	Death
[13]	49, M, African American	None	Bronchial washing cultures with a positive DNA probe	Bilateral miliary infiltrates with bilateral pleural effusions	Fluconazole	Clinical resolution
[29]	35, M, Samoan	Congenital deafness, hypertension, and asthma	IgM +, IgG+	Diffuse bilateral fine nodular reticular pattern	Itraconazole, amphotericin - > voriconazole	Full recovery
[30]	59, M, Caucasian	Diabetes	IgM +	Diffuse reticulonodular pattern, pulmonary micronodularity.	Amphotericin and fluconazole	Death
[31]	65, M, Hispanic	Diabetes, COVID, on steroids.	Serum β-d-glucan >500 pg/mL, tissue cultures	Miliary pattern with pulmonary nodules	Amphotericin	Death
[32]	61, M, Hispanic	HIV (Human Immunodeficiency Virus)	Autopsy, histopathology, cultures	Diffuse micronodular pattern	IV steroids	Death
[33]	52, M, Latinx	Diabetes, Chronic Kidney Disease treated with steroids	IgM +, IgG+	Diffuse miliary pulmonary nodules	Fluconazole, amphotericin, and IV steroids	Death
[33]	49, M, Latinx	COVID treated with steroids	IgM +, IgG+	Bilateral miliary lung nodules	Amphotericin and IV steroids	Clinical resolution
Present Case	46, F^a^, Latinx	Diabetes	IgM+, IgG, complement fixation	Bilateral miliary lung nodules	Fluconazole	Clinical improvement at 1 month

a Patient was born with XY genotype but identifies and presents as female due to androgen insensitivity syndrome, so will include as female for purposes of this study.

**Table 2:** Summary of Cases of Miliary Coccidioidomycosis in the Literature (n = 33 cases).

Characteristic	N (%)	
Year of Reported Case		
1999 and before	19/33	58 %
2000–2022	18/33	55 %
Male Sex	26/33	79 %
Female Sex	07/33	21 %
Age, mean	45.33 years	
HIV/AIDS diagnosis	03/33	9 %
Risk Factors for Disseminated Disease		
Diabetes	06/33	18 %
Pregnancy	04/33	12 %
Steroid use	04/33	12 %
Cancer/Malignancy	06/33	18 %
Medications associated with Neutropenia	01/33	3 %
Transplant (renal, lung, etc.)	01/33	3 %
COPD/ILD	02/33	6 %
IV drug use (IVDA)	02/33	6 %
ETOH	02/33	6 %
Tobacco Use Disorder	03/33	9 %
COVID	02/33	6 %
Other	03/33	9 %
Treatment with antifungals	N = 29	
Amphotericin	24/29	83 %
Fluconazole	9/29	31 %
Voriconazole	01/29	3.4 %
Ketoconazole	03/29	10 %
Steroids + Antifungal	08/29	27.5 %
Steroids (only)	03/33	9 % (of total patients)
Mortality	16/33	48 %

The approach of diagnosis does not differ depending on the primary or disseminated disease. The gold standard continues to be a high degree of suspicion based on anamnesis and serological identification through antibody testing, and/or identification of *Coccidioides* spp in culture mediums and/or demonstrating the characteristic spherules on histology in biopsy specimens.

A miliary pattern on chest imaging is not specific for a disease or pathogen and may be seen in metastatic cancer as well as several infectious organisms, including *Mycobacterium tuberculosis* and other fungi, most commonly from *Coccidioides* and *Histoplasma* [16]. The miliary pattern reflects the seeding of the pulmonary capillary beds by some matter, most often cancer cells or infectious organism with such propensities, such as mycobacteria and fungi.

The type and duration of antifungal treatment have not been standardized. Historically, treatment for primary coccidioidomycosis was only observation before the arrival of oral azoles. Nowadays, treatment is usually administered to patients who have more severe disease, require admission to the hospital, and/or those with risk factors for severe disease or dissemination, including diabetes mellitus, immunosuppression, pregnancy, and extreme ends of age [17]. Studies have shown that while amphotericin B was found to be efficacious for the treatment of coccidioidomycosis, its adverse effects, including kidney injury, electrolyte (potassium and magnesium) depletion, and infusion-related reactions, were considered too great for routine use, except for the more severe infections [[18], [19], [20]]. The azole anti-fungal class, which are significantly better tolerated and has a bio-available oral option for home use (such as for our patient), have been increasingly used in the more modern era. Our patient received fluconazole monotherapy and had a robust clinical response during her hospitalization and on initial follow-up.

## Conflict of interest

There are none.

## CRediT authorship contribution statement

**Oscar E. Gallardo-Huizar:** Writing – review & editing, Writing – original draft, Supervision. **Joyce Lee:** Writing – original draft, Methodology, Investigation. **Kailyn Kim:** Writing – original draft, Supervision. **Arthur C. Jeng:** Supervision.
