# Supplementary material for: Miliary coccidioidomycosis mimicking tuberculosis: Case report and review of literature
Source: Med Mycol Case Rep. 2024 Sep 13;46:100668. doi: 10.1016/j.mmcr.2024.100668 (PMC11418129; doi:10.1016/j.mmcr.2024.100668)
Supplement: Multimedia component 1 [file mmc1.docx]

Table 1: Reported Cases of Miliary Coccidioidomycosis in Adults

| Author and Year | Age/Gender/Race | Comorbidities Present at Diagnosis  (Co-existing Dx) | Diagnosis | Initial Chest Radiograph | Treatment | Outcome |
| --- | --- | --- | --- | --- | --- | --- |
| [21] | 37, F, Caucasian | Pregnant, myelofibrosis, on steroid therapy | Autopsy, histopathology, cultures | Fine miliary densities | None | Death |
| [22] | 55, M, White | None | Serum serology, serum complement fixation.  Definitive dx w/ urine and sputum cultures | Bilateral, 1-2mm nodular infiltrates distributed evenly throughout lung fields | IV and intrathecal amphotericin B | Alive |
| [23] | 35, M, Caucasian | Diabetes | Precipitin antibody C. immitis | Miliary pattern and pleural effusion | Amphotericin | Death |
| [24] | 23, M, White | None | Sputum cultures, serum complement fixation | Bilateral patchy areas of consolidation; multiple nodular radiopacities | IV amphotericin B, oral ketoconazole | Alive (“not returned to his usual state of health”) |
| [24] | 26, M, White | None | Sputum cultures, serum complement fixation | Bilateral patchy areas of consolidation; multiple nodular radiopacities | IV amphotericin B | Alive (“has returned to his usual state of health”) |
| [25] | 83, M, White | Chronic lymphocytic lymphoma | Blood culture | Miliary | Dexamethasone 24 mg | Death (one month later) |
| [25] | 20, M, Mexican American | Renal Transplant | Blood culture | Miliary with LLL infiltrate | Amphotericin B 590mg, and Prednisone 25mg | Alive (one month later) |
| [25] | 81, M, White | Chronic lymphocytic leukemia | Blood culture | Miliary | Amphotericin B 132 mg, Prednisone 10mg | Death (one month later) |
| [25] | 54, F, White | Breast carcinoma, Chronic lung disease | Blood culture | Miliary and LUL cavitary lesions | Amphotericin B (300mg), Prednisone 5 mg | Death (one month later) |
| [25] | 68, M, White | Large cell carcinoma of the lung, squamous cell carcinoma of the hypopharynx | Blood culture | Miliary | Dexamethasone (16mg) | Death (one month later) |
| [25] | 24, M, White | AIDS | Blood culture | Miliary | Amphotericin B (1600mg), ketoconazole | Alive (one month later) |
| [25] | 62, M, White | Chronic lung disease, idiopathic cardiomyopathy | Blood culture | Miliary | Amphotericin B (1040 mg), prednisone 25mg | Death (one month later) |
| [25] | 71, M, White | Glioblastoma | Blood culture | Miliary | Amphotericin B (155mg), dexamethasone 12 mg | Death (one month later) |
| [25] | 32, M, Black | AIDS | Blood culture | Miliary | Ketoconazole | Death (one month later) |
| [9] | 38, M, African American | Hypertension, CHF, cocaine abuse, EtOH, tobacco | Complement fixation | RML infiltrate (on initial chest radiograph), subsequent imaging with miliary pattern | Fluconazole | Death |
| [9] | 23, M, Caucasian | IVDA, tobacco, EtOH | Complement fixation | Miliary pattern | Fluconazole and amphotericin | Death |
| [9] | 65, M, Caucasian | None | Complement fixation | Miliary pattern | Fluconazole and amphotericin | Alive |
| [9] | 41, F, Caucasian | IVDA, tobacco | Complement fixation | Miliary pattern | Fluconazole | Alive |
| [9] | 26, M, African American | None | Complement fixation | Miliary pattern | Fluconazole, amphotericin, interferon | Death |
| [9] | 34, M, Caucasian | None | Complement fixation | LL lobar infiltrate (on initial chest radiograph), subsequent imaging with miliary pattern | Amphotericin | Alive |
| [9] | 25, F, Hispanic | Pregnant | Complement fixation | Miliary pattern | amphotericin | Alive |
| [9] | 27, F, Hispanic | Pregnant | Complement fixation | Miliary pattern | Fluconazole and amphotericin | Alive |
| [26] | 21, F, Hispanic | Gestational Diabetes and Pregnancy | Blood cultures, complement fixation | Diffuse miliary infiltrates | Amphotericin, fluconazole, steroids | Clinical Improvement |
| [27] | 31, M, Korean | adrenoleukodystrophy | skin biopsy with spherules | Miliary infiltrates | Amphotericin, fluconazole | Clinical resolution |
| [28] | 78, M, Caucasian | Rheumatoid Arthritis on Infliximab therapy | BAL, endospores visualized | Bilateral miliary interstitial infiltrate pattern | amphotericin | Death |
| [13] | 49, M, African American | None | Bronchial washing cultures with a positive DNA probe | Bilateral miliary infiltrates with bilateral pleural effusions | Fluconazole | Clinical resolution |
| [29] | 35, M, Samoan | Congenital deafness, hypertension, and asthma | IgM +, IgG+ | Diffuse bilateral fine nodular reticular pattern | Itraconazole, amphotericin -> voriconazole | Full recovery |
| [30] | 59, M, Caucasian | Diabetes | IgM + | Diffuse reticulonodular pattern, pulmonary micronodularity. | Amphotericin and fluconazole | Death |
| [31] | 65, M, Hispanic | Diabetes, COVID, on steroids. | Serum β-d-glucan >500 pg/mL, tissue cultures | Miliary pattern with pulmonary nodules | Amphotericin | Death |
| [32] | 61, M, Hispanic | HIV (Human Immunodeficiency Virus) | Autopsy, histopathology, cultures | Diffuse micronodular pattern | IV steroids | Death |
| [33] | 52, M, Latinx | Diabetes, Chronic Kidney Disease treated with steroids | IgM +, IgG+ | Diffuse miliary pulmonary nodules | Fluconazole, amphotericin, and IV steroids | Death |
| [33] | 49, M, Latinx | COVID treated with steroids | IgM +, IgG+ | Bilateral miliary lung nodules | Amphotericin and IV steroids | Clinical resolution |
| Present Case | 46, F*, Latinx | Diabetes | IgM+, IgG, complement fixation | Bilateral miliary lung nodules | Fluconazole | Clinical improvement at 1 month |

*Patient was born with XY genotype but identifies and presents as female due to androgen insensitivity syndrome, so will include as female for purposes of this study.

Table 2: Summary of Cases of Miliary Coccidioidomycosis in the Literature (n= 33 cases)

| Characteristic | N (%) |
| --- | --- |
| Year of Reported Case |  |
| 1999 and before | 19/33 58% |
| 2000 - 2022 | 18/33 55% |
| Male Sex | 26/33 79% |
| Female Sex | 07/33 21% |
| Age, mean | 45.33 years |
| HIV/AIDS diagnosis | 03/33 9% |
| Risk Factors for Disseminated Disease |  |
| Diabetes | 06/33 18% |
| Pregnancy | 04/33 12% |
| Steroid use | 04/33 12% |
| Cancer / Malignancy | 06/33 18% |
| Medications associated with Neutropenia | 01/33 3% |
| Transplant (renal, lung, etc.) | 01/33 3% |
| COPD/ILD | 02/33 6% |
| IV drug use (IVDA) | 02/33 6% |
| ETOH | 02/33 6% |
| Tobacco Use Disorder | 03/33 9% |
| COVID | 02/33 6% |
| Other | 03/33 9% |
| Treatment with antifungals | N = 29 |
| Amphotericin | 24/29 83% |
| Fluconazole | 9/29 31% |
| Voriconazole | 01/29 3.4% |
| Ketoconazole | 03/29 10% |
| Steroids + Antifungal | 08/29 27.5% |
| Steroids (only) | 03/33 9% (of total patients) |
| Mortality | 16/33 48% |

References:

9. E.L. Arsura, W.B. Kilgore, Miliary coccidioidomycosis in the immunocompetent, Chest. 117 (2000) 404–409.

13. D. Sotello, M. Rivas, A. Fuller, T. Mahmood, M. Orellana-Barrios, K. Nugent, Coccidioidomycosis with diffuse miliary pneumonia, Proc.29 (2016) 39–41.

21. J.J. Castellot, R.L. Creveling, F.W. Pitts, Fatal miliary coccidioidomycosis complicating prolonged prednisone therapy in a patient with myelofibrosis, Ann. Intern. Med. 52 (1960) 254–258.

22. H.W. Randle, Miliary coccidioidomycosis, Ariz. Med. 32 (1975) 408–410.

23. E. Goldstein, Miliary and disseminated coccidioidomycosis, Ann. Intern. Med. 89 (1978) 365–366.

24. R.A. Larsen, J.A. Jacobson, A.H. Morris, B.A. Benowitz, Acute respiratory failure caused by primary pulmonary coccidioidomycosis. Two case reports and a review of the literature, Am. Rev. Respir. Dis. 131 (1985) 797–799.

25. N.M. Ampel, K.J. Ryan, P.J. Carry, M.A. Wieden, R.B. Schifman, Fungemia due to Coccidioides immitis. An analysis of 16 episodes in 15 patients and a review of the literature, Medicine. 65 (1986) 312–321.

26. N.F. Crum, G. Ballon-Landa, Coccidioidomycosis in pregnancy: case report and review of the literature, Am. J. Med. 119 (2006) 993.e11–7.

27. D.W. Park, J.W. Sohn, H.J. Cheong, W.J. Kim, M.J. Kim, J.H. Kim, C. Shin, Combination therapy of disseminated coccidioidomycosis with caspofungin and fluconazole, BMC Infect. Dis. 6 (2006) 26.

28. M.P. Rogan, K. Thomas, Fatal miliary Coccidioidomycosis in a patient receiving infliximab therapy: a case report, J. Med. Case Rep. 1 (2007) 79.

29. A. Dev, D. Janysek, J. Gnecco 4th, K. Haghayeghi, Disseminated Coccidioidomycosis Following Insufficient Treatment at Initial Presentation: Case Report, J Investig Med High Impact Case Rep. 8 (2020) 2324709620949315.

30. V.R. Wormser, Z.Y. Li, R. Libke, E. Rahmati, Miliary pattern pulmonary infiltrates in a diabetic patient: Coccidioidomycosis, IDCases. 22 (2020) e00977.

31. J.C. Chen, D. Wong, S. Rabi, S. Worswick, B. DeClerck, J. Gibb, All That Coughs Is Not COVID-19: A Delayed Diagnosis of Disseminated Coccidioidomycosis Following Severe Acute Respiratory Syndrome Coronavirus 2 Infection, Open Forum Infect Dis. 8 (2021) ofab246.

32. O. Aduroja, J. Okudo, A. Padilla, Disseminated Coccidioidomycosis Presenting as Septic Shock with Multiorgan Failure, Case Rep. Infect. Dis. 2021 (2021) 8837493.

33. R. Sous, Y. Levkiavska, R. Sharma, R. Jariwal, D. Amodio, R.H. Johnson, A. Heidari, R. Kuran, Two Cases of Miliary and Disseminated Coccidioidomycosis Following Glucocorticoid Therapy and Literature Review, J Investig Med High Impact Case Rep. 10 (2022) 23247096211051930
